# Verteporfin inhibits gastric cancer cell growth by suppressing adhesion molecule FAT1

**DOI:** 10.18632/oncotarget.21946

**Published:** 2017-10-19

**Authors:** Myoung-Hee Kang, Gi Seok Jeong, Duane T. Smoot, Hassan Ashktorab, Chang Mo Hwang, Byung Sik Kim, Hee Sung Kim, Yun-Yong Park

**Affiliations:** ^1^ Asan Institute for Life Sciences, Asan Medical Center, Seoul, Republic of Korea; ^2^ Department of Convergence Medicine, University of Ulsan College of Medicine, Seoul, Republic of Korea; ^3^ Biomedical Engineering Research Center, Asan Institute for Life Sciences, Asan Medical Center, Seoul, Republic of Korea; ^4^ Department of Internal Medicine, Meharry Medical College, Nashville, TN, USA; ^5^ Department of Medicine and Cancer Center, Howard University, Washington, DC, USA; ^6^ Department of Gastric Surgery, University of Ulsan College of Medicine, Asan Medical Center, Seoul, Republic of Korea

**Keywords:** verteporfin, gastric cancer, FAT1, metastasis

## Abstract

Gastric cancer (GC) is a leading cause of death worldwide and in urgent need of targeted drug development. In the current, we investigated the ability of a repositioned drug verteporfin (VP), originally a treatment for macular degeneration, to inhibit GC cell growth. VP inhibited growth of various GC cell lines. Gene expression profiling of GC cell lines treated with VP revealed that migration-related genes and those with oncogenic potential were down-regulated. Of these genes, we found that FAT1, an adhesion molecule promoting cell invasion, was highly suppressed by VP. Silencing of FAT1 suppressed cell migration and invasion as VP did. FAT1 expression was up-regulated in tumors, and patients with high FAT1-expressing tumors had a worse prognosis. We propose that VP- targeting FAT1 to suppress metastatic potential is a promising therapeutic strategy against GC.

## INTRODUCTION

Gastric cancer (GC) is the predominant lethal cancer in Asia [6]. Traditionally, the standard treatment in cases of early GC (stage I and II) is surgical resection, often accompanied by adjuvant chemotherapy [8]. Although recently, targeted molecular therapeutics such as trastuzumab directed against the HER2 receptor and bevacizumab against vascular endothelial growth factor (VEGF)-A have been developed for and used successfully to treat certain cancers, there are none with more than limited efficacy in GC [22], suggesting the urgent need to identify new molecular targets for this disease [5], [23].

Verteporfin (VP), a drug widely used to treat macular degeneration, was recently identified through a drug repositioning strategy as an agent for the treatment of cancer [11]. VP acts as a transcriptional inhibitor of the direct interaction between YAP1 and TEAD, suppressing the oncogenic activity of the YAP1-TEAD complex [11]. Studies of a mouse model of liver cancer and an *in vitro* colon cancer model suggest that VP has efficacy for the treatment of the cancer [11], [25]; however, the mechanism by which VP inhibits cancer growth is poorly understood and VP has not so far been investigated for any therapeutic effects in GC.

Originally used to eliminate the abnormal blood vessels that occur with macular degeneration [19], VP accumulates in these vessels and, when stimulated by non-thermal red light at 689 nm, produces highly reactive short-lived singlet oxygen, resulting in local damage to the endothelium and blockage of the vessels [10].

Tumor metastasis is the main cause of death in cancer patients [1]. In GC, lymph node (LN) metastasis is common and the lymphatic vessels are an important route [1]. Since VP can eliminate blood vessel formation, it is speculated that VP could be used to suppress tumor progression by affecting the lymphatic vessels via which cells metastasize [2].

In the current study, we investigated VP as a potential therapeutic agent for GC. We demonstrate using various assays, including human organoids, a xenografted mouse model, and a 3D microfluidic channel system, that VP inhibits GC cell proliferation, cell migration, and invasion. Gene expression profiling of GC cell lines after treatment with VP revealed that FAT Atypical Cadherin 1 (FAT1) expression was diminished and therefore FAT1 is a potential target of VP. FAT1 expression is up-regulated in GC tumors from patients and is associated with a bad prognosis. Furthermore, FAT1 expression is associated with cell migration and invasion. Our study provides evidence that VP is a potential therapeutic agent for GC targeting FAT1, and that FAT1 is a potential therapeutic target for the treatment of GC.

## RESULTS

### Verteporfin inhibits GC cell proliferation

**Figure 1:**
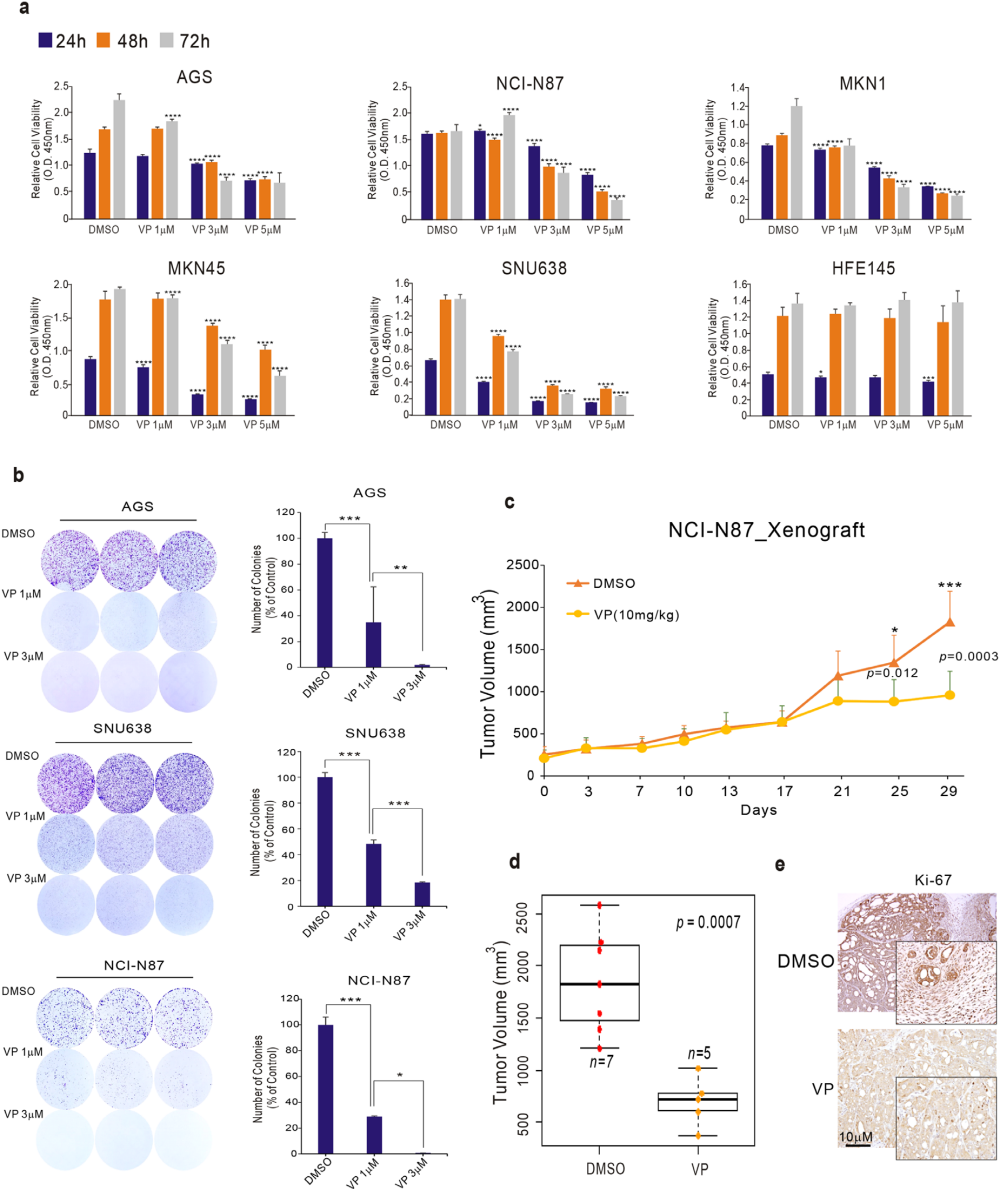
Effect of VP on GC cell growth **(a)** Cell viability assay and **(b)** colony-formation assays after VP treatment of the indicated GC cell lines. **(c**–**d)** NCI-N87 GC cells were injected into nude mice with or without VP, and tumor volume was measured at the indicated time points. Mice were sacrificed and tumor volumes were measured. **(e)** Immunohistochemical analysis of the xenografts. Data represent the mean ± standard deviation from three independent replicates (cell line data) (^*^p<0.05, ^**^p<0.01, ^**^p<0.005, ^****^p<0.001 by Student's t-test).

To investigate whether VP is effective against GC, we first performed cell proliferation assays and colony-forming assays on GC cell lines AGS, NCI-N87, MKN1, MKN45, and SNU638 treated with VP. VP efficiently inhibited cell proliferation in a time- and dose-dependent manner (Figure 1a). VP did not inhibit cell proliferation of the normal gastric epithelial cell line HFE145, suggesting that VP is selectively effective on GC cell lines (Figure 1a). Colony-forming ability was significantly decreased in the VP-treated GC cell lines (Figure 1b). To investigate the efficacy of VP *in vivo*, we treated mice bearing NCI-N87 xenografts with VP. When tumors reached an average size of 100mm^3^, treatment was then initiated with VP (10mg/kg) injected intraperitoneally at 2 day intervals for 3weeks. Consistent with observations *in vitro*, tumor volume and tumor weight but not body weight was significantly reduced upon treatment with VP (Figure 1c, 1d and Supplementary Figure 1). That proliferation was reduced by treatment with VP in the xenograft samples was confirmed by examining expression of the cell proliferation marker Ki67 by IHC (Figure 1e).

Taken together, these results clearly demonstrate that VP has a crucial role in the growth and progression of gastric tumors, and, importantly, indicate that VP could be a therapeutic agent for the treatment of GC.

### FAT1 is a target of VP in GC

**Figure 2:**
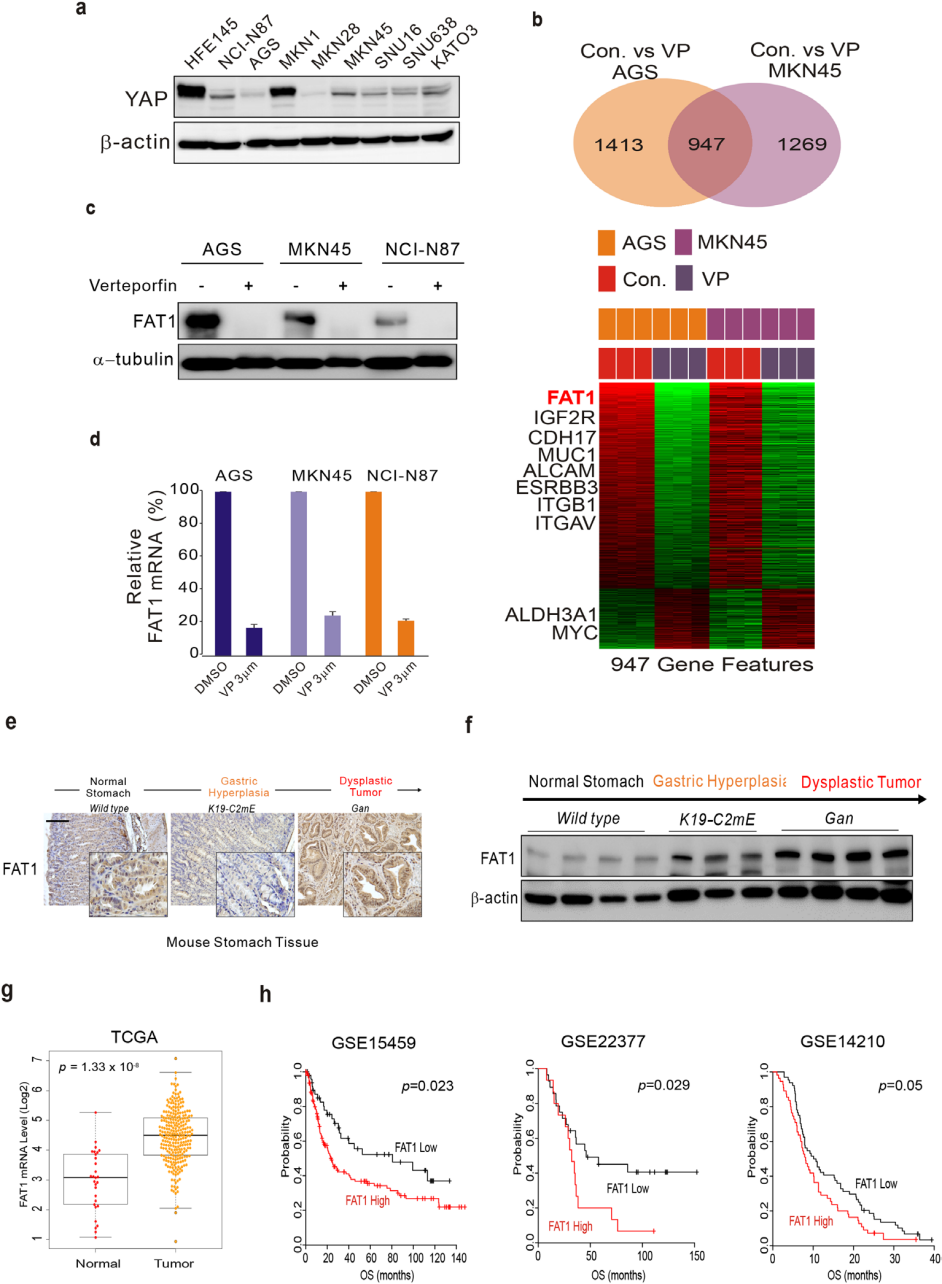
FAT1 is a target of VP in GC **(a)** YAP1 expression in GC cells. Western blot analysis of lysates from the indicated GC cell lines. **(b)** Gene expression profile. Microarray analysis of AGS and MKN45 GC cell lines in the absence (control) and presence of VP. Venn diagram of genes with significantly different expression between the control and VP-treated groups of two GC cell lines. A univariate test (two-sample *t*-test) with a multivariate permutation test (10,000 random permutations) was applied. In each comparison, we applied a cut-off *p*-value of <0.001 to retain genes with expression that differed significantly between the two groups of cells. Expression patterns of selected genes shared in the two cell lines. The expression of only 947 genes was commonly up- or down-regulated in all three cohorts. Colored bars at the top of the heat map represent samples as indicated. **(c)** Protein expression by Western blotting and **(d)** mRNA expression by qRT-PCR experiment from cells treated with VP or DMSO (control). **(e)** Immunohistochemical staining of FAT1 in wild-type (left), K19-C2mE (middle), and Gan (right) mouse stomachs. **(f)** WB analysis from gastric cancer mouse model used in Figure (2e). **(g)** FAT1 expression in GC patient cohorts. **(h)** Patients in the indicated GC cohort from GEO were dichotomized according to expression of FAT1 and patients with relatively high or low expression of FAT1 and was considered for plotting. Data represent the mean ± standard deviation from the indicated samples. A Student's *t*-test was applied to examine differences for significance.

Since VP functions as a disruptor of the interaction between YAP1 and TEAD1 [11], we screened expression levels of YAP1 across GC cell lines to see whether the anti-proliferation effect of VP is dependent on YAP1. Western blotting demonstrated that YAP1 expression was very diverse among the cell lines (Figure 2a). Although YAP1 expression was relatively low in AGS, MKN28, and SNU638, VP was able to inhibit proliferation of those cells. By contrast, YAP1 expression was very high in the normal gastric epithelial cell line, HFE145; however, VP did not inhibit HFE145 cell growth, suggesting that the anti-proliferation effect by VP is YAP1-independent.

Since VP has an anti-proliferative effect regardless of YAP1 expression, we investigated other signaling molecules that may be involved in VP inhibition of GC cell proliferation. Thus, we generated gene expression profiles of VP-treated and untreated AGS and MKN-45 GC cell lines to investigate which genes are influenced by VP. Gene expression profiling revealed that expression of 947 genes was significantly altered between the control (untreated) and VP-treated groups of the two cell lines. A Venn diagram of the genes with significantly different expression is shown (Figure 2b), which highlights that a substantial number are known downstream targets of VP. Among the 947 genes, we found out FAT1 [27], IGF2R [17], CDH17 [12], MUC1 [18], ALCAM [24], ITGB1 [7], ERBB3 and ITGAV are involved in tumorigenesis. Of these, FAT1 was highly down-regulated in the presence of VP. To validate the gene expression profile data, we performed western blots and qRT-PCR assays. As shown in Figure 2c and 2d, FAT1 including other gene's expression (Supplementary Figure 2) was decreased or increased by VP treatment. Interestingly, YAP, which is well known target of VP, expression was not much changed by VP treatment (Supplementary Figure 3). Since FAT1 function in GC is poorly understood, we examined FAT1 expression in GC cell line, mouse model and tumor samples from GC patients. FAT1 was expressed in GC cell lines including normal gastric cell, HFE145 (Supplementary Figure 4). Using mouse GC model [14], we show that FAT1 expression, as determined by western blotting and qRT-PCR, gradually increased during tumorigenesis (Figure 2e and 2f), demonstrating that FAT1 has oncogenic potential. Using gene expression profile from gastric cancer patients, we performed genomic analysis. TCGA is the best well known data platform form National Cancer Institute (NCI). TCGA gastric cancer data set was composed of 271 cancer tissues and 25 normal tissues. Gene expression profile reveals that FAT1 expression was up-modulated in cancer tissues compared with normal tissues (Figure 2g and Supplementary Figure 5). Next, we performed survival analysis correlating FAT1 expression with overall survival duration. We used GEO (Gene expression omnibus) public database. GSE15459, SGE23377 and GSE14210 data set was from gastric cancer patients after surgery and found that patients with tumors highly expressing FAT1 had a worse prognosis (Figure 2h).

Our data suggest that FAT1 is the target of VP that confers the drug's anti-proliferative property.

### VP inhibits cell migration and invasion in GC

**Figure 3:**
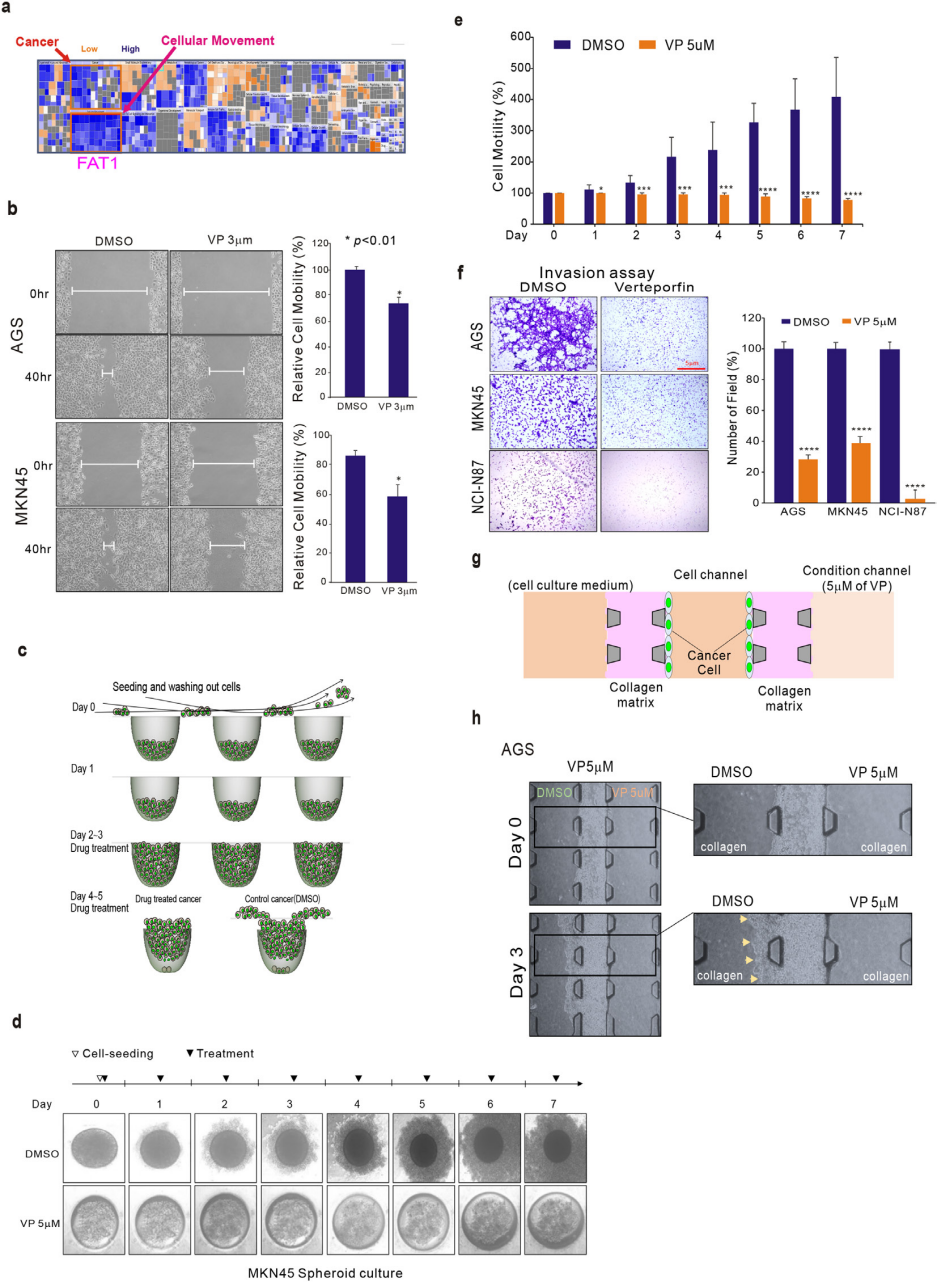
VP suppression of GC cell migration and invasion **(a)** VP targets FAT1 signaling. Ingenuity Pathway Analysis (IPA) of the genes differentially expressed after VP treatment. **(b)** Effect of VP (3 μM) treatment on the migration of AGS and MKN45 cell lines assessed using the wound healing assay. **(c)** Schematic diagram of cell seeding and outgrowth from the concave microwell migration system. **(d)** After seeding, cells that did not settle were gently flushed out (day 0). Processes for culturing MKN45 growing in the concave microwell arrays (day 1). After day 1, VP (5 μM) or DMSO was added to the medium (day 3). This was repeated every other day. **(e)** Plot of the invading cell numbers. **(f)** Conventional cell invasion assay. Cells (1 × 10^5^ cells/well) were seeded in the upper chamber, which was coated with Matrigel, and growth medium containing VP (5 μM) was added to the lower chamber. After 48 h of incubation, the cells that invaded the lower surface of the insert were stained with Diff-Quik stain and counted by microscopy. **(g)** A schematic illustration of the microfluidic device showing the growing and spreading of AGS cells in the microwell. **(h)** Microscope images of AGS cell migration into the 3D collagen matrix of the microfluidic device. Bars indicate the mean ± standard deviation per group from three different experiments.

We used the gene expression profile data from the untreated and VP-treated AGS and MKN-45 GC cell lines to analyze the pathways involved in VP treatment. Ingenuity Pathway Analysis (IPA) results revealed that genes associated with cellular movement, which is correlated with tumor metastasis, were down-regulated by VP treatment (Figure 3a). FAT1, which was highly down-regulated by VP, is involved in cell migration and invasion pathways, and is known to function as an adhesion molecule that contributes to cancer cell survival and migration [27]. Our investigation also confirmed that silenced-FAT1 influenced cell proliferation in GC cells (Supplementary Figure 6). Blood vessel formation is a major survival pathway via which cancer cells can escape from primary organ sites [1]. That VP, known for its role as an inhibitor of blood vessels in the treatment of macular degeneration, caused the down-regulation of genes associated with cell migration and movement is in keeping with the notion that VP would be effective at inhibiting tumor metastasis.

Since VP treatment influenced the expression of genes associated with cell migration and invasion, we examined the potential association of VP in assays of cancer cell migration and invasion. We performed wound healing assays with AGS and MKN45 GC cell lines after treatment with VP, or with DMSO as a control. Cell mobility was significantly diminished in VP-treated GC cells compared with DMSO-treated cells (Figure 3b). In addition, PPIX [11], which has similar pharmacological activity of VP, also influenced cell invasion as VP did (Supplementary Figure 7). We used a recently developed concave microwell system [4], [9] to further examine cell proliferation and migration. As depicted in Figure 3c, the MKN45 cells seeded in the concave well tended to spread out into the space available, mimicking metastatic behavior *in vivo*. In the same concave well system, we examined whether VP inhibited AGS GC cell migration. While the cells spread out in the concave well as expected, VP-treated cells failed to migrate to other areas (Figure 3d). To quantify whether VP significantly inhibited cell migration, we measured the area of cell spread in the absence of VP, which was interpreted as a cell motility and migration index (Figure 3e). Next, we performed a conventional invasion assay, which examines cell passage through a matrigel-coated chamber, to assess the anti-invasive effect of VP on GC cells. As expected, VP significantly inhibited cell invasion across the matrigel-coated surface (Figure 3f). To more precisely examine invasion inhibition by VP, we performed cell invasion assays using a 3D micro-fluidic channel system, which reflects *in vivo* metastasis conditions. As shown in Figure 3g, the AGS GC cells were able to invade other areas by disrupting the collagen matrix, thereby reflecting metastatic behavior and conditions *in vivo* (Figure 3g). After cell seeding into the microwells, in DMSO control medium the area the AGS cells adhered to increase over time, but in the presence of VP there was no evidence of cell invasion (Figure 3h). Taken together these results indicate that VP is effective at inhibiting cell migration and metastatic-like cell behavior.

### Function of FAT1 targeted by VP in GC

**Figure 4:**
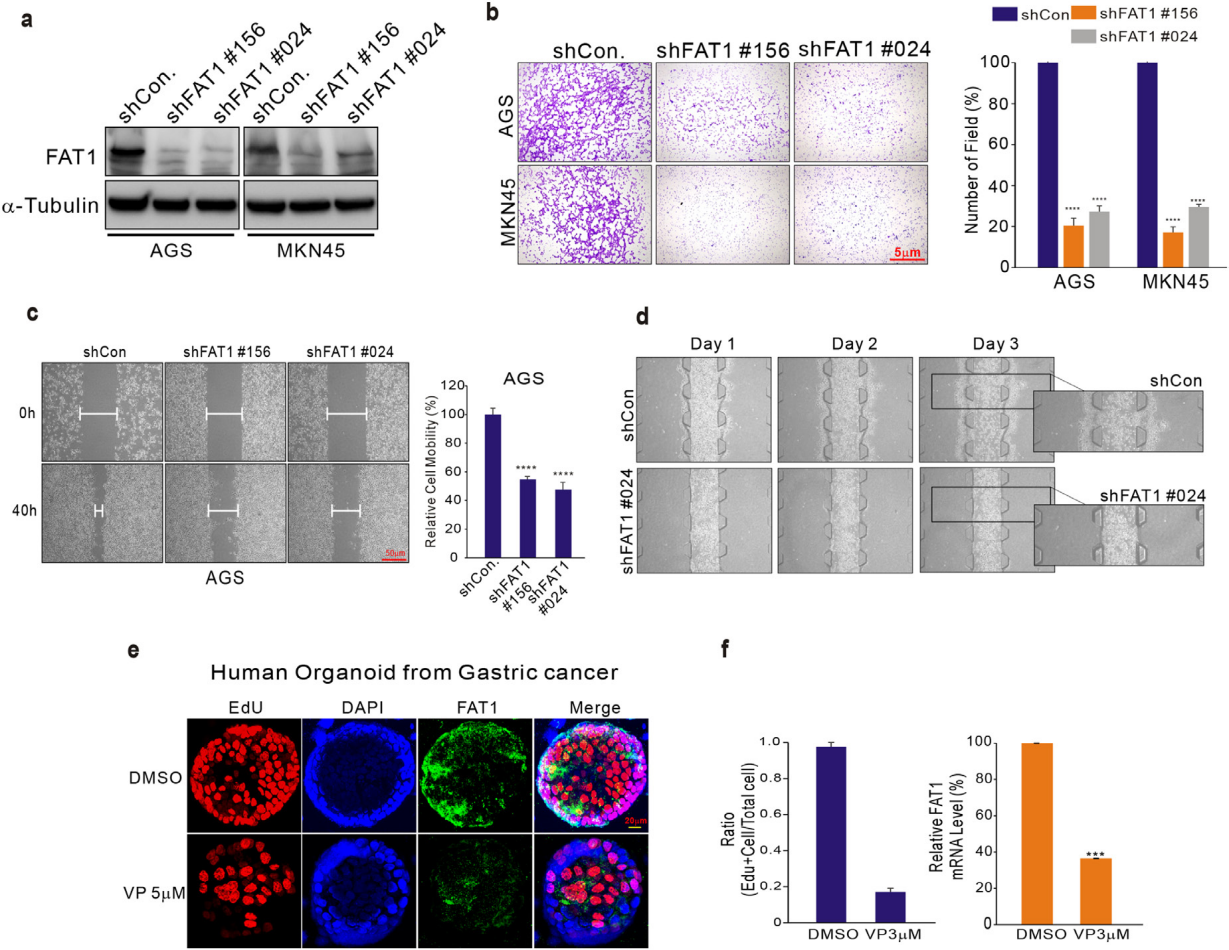
Expression of FAT1 in GC specimens **(a–d)** AGS cells were transduced with shCon or shFAT1 to examine the effect of down-regulating FAT1. (a) Transduced cells were harvested for Western blotting and 48 hours after transduction evaluated for migration and invasive ability using wound healing (b-c) and concave microwell array (d) assays. **(e)** Organoids derived from human gastric fundus cancer samples. Organoids were immunostained with 5-ethynyl-2’-deoxyuridine (EdU; red), FAT1 (green), and Hoechst 33342 (cell nuclei; blue). **(f)** Left, total cell number and EdU-labeled nuclei were counted in five organoids and expressed as a ratio of EdU-positive cells/total cells. Right, FAT1 expression was measured by qPCR in organoids cultured in DMSO or VP (3 μM).

Gene expression data revealed that FAT1 is dominantly suppressed by VP treatment (Figure 2). To investigate the function of FAT1 in human cancer, we performed a variety of experiments. First, we performed invasion assays on AGS GC cells in which FAT1 had been silenced by inhibition with shRNA (Figure 4a). The results clearly show that silencing of FAT1 significantly inhibited cell invasion to the same degree as VP treatment (Figure 4b). In addition, in the cell-FAT silenced, cell migration was also decreased compared with control cell (Figure 4c and Supplementary Figure 8). Next, we performed wound healing and microfluidic chip assays; these also demonstrated decreased cell migration of AGS GC cells bearing silenced FAT1 (Figure 3c and 3d). Recently, human organoid culture systems have been developed that allow the three-dimensional growth of cells, thus retaining their tissue identity [21]. Next, we tested the effects of VP in a GC organoid model using tumor tissue from GC patients. Compared to untreated organoids, those treated with VP had a significantly decreased proliferation index, as determined by the number of Edu-positive cells (Figure 4e, 4f and Supplementary Figure 9a). In addition, FAT1 but not YAP1 expression was also decreased in the VP-treated organoids (Figure 4f and Supplementary Figure 9b), since FAT1 is a VP-target, suggests that VP has a therapeutic effect in GC.

Our data clearly suggest that FAT1 has oncogenic potential, and could therefore be a therapeutic target in GC.

## DISCUSSION

VP was originally identified as a disruptor of the interaction between TEAD and YAP [11], which are potent oncogenic factors in many cancers, and while it has been shown to inhibit liver cancer formation [25], the cancer suppressive mechanisms of VP require elucidation. Recent studies demonstrate that growth inhibition after VP treatment is independent of YAP1 expression or that of the related Hippo effector TAZ in colon cancer [25], which is in line with our finding that YAP1 expression did not correlate with sensitivity to VP in GC cell lines.

Gene expression profiles of VP-treated GC cell lines revealed that cancer-associated genes such as FAT1 [27], IGF2R [17], CDH17 [12], MUC1 [18], ALCAM [24], ITGB1 [7], ERBB3 and ITGAV were altered, suggesting that VP suppresses oncogenic potential. Genomic analysis has identified that FAT1 is a tumor suppressor and mutation of FAT1 leads to human cancer [13]. However, FAT1 may also function as an oncogenic protein since FAT1 promotes tumor migration via induction of actin polymerization, leading to loss of membrane localization, which is correlated with more aggressive tumors [27]. FAT1 expression is up-regulated in patients with leukemia and pre B-acute lymphoblastic leukemia [27], suggesting that FAT1 function is distinct depending on the cell context. In the present study, we found that loss of FAT1 resulted in decreased cancer cell growth, and that FAT1 expression in tumor samples from GC patients and from a GC mouse model was highly up-regulated compared with that in normal gastric tissues. In addition, high FAT1 expression was clearly associated with a worse prognosis in GC patients. Thus, we suggest that targeting FAT1 in cases where it has oncogenic properties would be an efficient way to treat the GC patient.

For the cancer patient, tumor metastasis is the primary factor influencing mortality. Gene network analysis from expression profiles in VP-treated cells revealed that the genes involved in cell migration and invasion, key to the initial process driving tumor metastasis, were significantly decreased. Since it is well known that VP suppresses angiogenesis by inducing ROS, down-regulation of genes involved in cell migration and invasion is in keeping with this intrinsic function, and suggests that VP would be effective at blocking angiogenesis during tumor metastasis.

Our data demonstrate that FAT1 functions as an oncogene by influencing cell adhesion, resulting in cancer cell growth promotion although FAT1 function is controversial. Further studies are now required to elucidate the mechanisms by which FAT1 contributes to tumor metastasis in GC.

In the current study, we have demonstrated that VP, a benzoporphyrin derivative that functions as a photosensitizer for photodynamic therapy to eliminate abnormal blood vessels in macular degeneration, also has a distinct anti-proliferative effect on GC cells without requirement for photostimulation. In summary, we have identified a potential role for VP as a therapeutic agent in GC and also suggest that the FAT1 expression is a useful prognostic biomarker for prognosis. Thus, targeting FAT1 could be a useful strategy for future GC treatment.

## MATERIALS AND METHODS

### Cell lines and reagents

GC cell lines (AGS, NCI-N87, MKN1, MKN45, and SNU638) were purchased from the American Type Culture Collection or Korean Cell line Bank. The normal gastric epithelial cell line, HFE145 was generated by Drs. Duane T Smoot and Hassan Ashktorab [26]. Cells were grown in Dulbecco's modified Eagle's essential medium (DMEM) or RPMI 1640 medium supplemented with 10% fetal bovine serum. Cells were incubated at 37°C in a humidified incubator with 5% CO_2_. VP (#1711461) was purchased from U.S. Pharmacopeial Convention (Rockville, MD, USA).

### Gene expression profiling

ASG or MKN45 cells were harvested for RNA isolation with or without treatment with VP for 48 hours. Total RNA was extracted using a mirVana miRNA isolation labeling kit (Ambion Inc., Waltham, MA), and 500 ng was used for labeling and hybridization (Human BeadChip V4 microarray, Illumina, San Diego, CA) according to the manufacturer's protocols. After the bead chips were scanned with an Illumina BeadArray Reader, the microarray data were normalized using the quantile normalization method in the Linear Models for Microarray Data package in R (www.r-project.org). The expression level of each gene was log_2_ transformed before further analysis. The microarray data generated in this study are available in the NCBI Gene Expression Omnibus public database (GSE78050).

### Gene expression data analysis

Gene expression data from NCBI GEO databases are publically available (accession numbers GSE13861, GSE26899, GSE29272, GSE62254). All the data were downloaded and analyzed using Biometric Research Branch (BRB)-ArrayTools software [28]. Gene expression profile using microarray was generated from indicated cells (Figure 2b) and the data was analyzed by Ingenuity Pathway Analysis (IPA) to know which pathway is associated with VP treatment (Figure 3a).

### Quantitative real-time RT-PCR

Total RNA was extracted from the indicated cell lines using a mirVana miRNA isolation kit (Ambion, Inc., Waltham, MA, USA) according to the manufacturer's instructions, and assayed using real-time quantitative RT-PCR (qRT-PCR) with TaqMan expression assay(Applied Biosystems) FAT1 (Hs00170627_m1) primers specific for each gene. qRT-PCR was performed using the StepOnePlus Real-Time PCR system with a 96-well block module (ABI). Cycling conditions were 45°C for 30 min and 95°C for 10 min, followed by 40 cycles of 95°C for 15 s and 60°C for 60 s. Relative amounts of mRNA were calculated from the threshold cycle number using the expression of cyclophilin A (PPIA;Hs99999904_m1) as a housekeeping control. All experiments were performed in triplicate and the values averaged.

### Wound healing assay and conventional (Matrigel) invasion assay

AGS or MKN45 cells were seeded at a density of 1 × 10^5^ cells/well in 12-well plates and pre-incubated for 24 h in serum-free RPMI (Invitrogen) before wounding the cell monolayer with a plastic tip. Cells were then grown in medium with or without various drugs in the absence or presence of VP. Cell migration into the wound surface was monitored by microscopy after 24 h and reported as the estimated ratio of the remaining wounded area relative to the initial wound area. Quantification of the closure of the monolayer was performed using ImageJ software and results are expressed as the percentage of wound closure. This assay was repeated three times independently. For the conventional invasion assays, 1 × 10^5^cells/well were seeded in the upper chamber, which was coated with Matrigel (Corning), and serum-free medium containing VP was added to the lower chamber. After 24 h, non-migrating cells were removed from the upper chamber with a cotton swab, and the cells on the lower surface of the insert were stained with Diff-Quik stain (Biochemical Sciences, Swedesboro, NJ); the invading cells were counted by microscopy. All experiments were repeated three times.

### Immunohistochemistry

Representative formalin-fixed, paraffin-embedded tissue sections (5 μm-thick) from GC patient tumor samples and mouse xenograft samples were examined by immunohistochemistry (IHC). Tissue sections were deparaffinized in xylene and rehydrated in a graded alcohol series. Antigen retrieval was performed by irradiation (microwave oven) for 20 min in a jar containing 0.01 M citrate buffer (pH 6.0). Endogenous peroxidase was blocked using 3% hydrogen peroxide in phosphate-buffered saline (PBS) for 15 min. The specimens were incubated with a protein-blocking solution consisting of PBS (pH 7.5) with 5% normal horse serum for 30 min at room temperature. Incubation with primary antibodies was performed at 4°C overnight. Primary antibodies included anti-FAT1 (A304-403A; Bethyl Laboratories Inc., Montgomery, TX, USA) diluted 1:250 and anti-Ki67 (ab833; Abcam, Cambridge, UK) diluted 1:50. The samples were then rinsed and incubated with peroxidase-conjugated anti-goat IgG for 1 h at room temperature. Next, the slides were rinsed with PBS and incubated for 5 min with an ImmPACT DAB Kit (VECTOR Laboratories, Burlingame, CA, USA). The sections were washed three times with distilled water, counterstained with Mayer's hematoxylin (Sigma-Aldrich, St Louis, MO), and washed once each with distilled water and PBS. Slides were mounted using a Universal Mount (VECTOR Laboratories) and examined using a bright field microscope. FAT1 and Ki67 expression in tumor cells was assessed by independent pathologists according to previously described methods.

### Xenograft experiments

Male athymic nude mice were purchased from Oriental Bio (Seoul, Korea) and maintained according to the Animal Experimentation Guidelines of the ASAN Medical Center. All mouse studies were approved and supervised by the ASAN Medical Center Institutional Animal Care and Use Committee (IACUC No. 2015-14-047). All animals used were 8–12 weeks of age at the time of injection. To establish tumors, cell (4 × 10^6^ cells in 50 μl of normal saline) were injected subcutaneously in the right dorsal flank injecting 50μl of cells at one time. For the VP treatment, VP was purchased from [U.S. Pharmacopeial Convention (USP)]. VP was dissolved in dimethyl sulfoxide (DMSO) was diluted using phosphate-buffered saline (PBS), and the mice were injected intraperitoneally with VP (10 mg/kg) every two days as described previously with minor modification [25]. VP treatment continued for 3 weeks following tumor cell injection. At the time of sacrifice, mouse weight, tumor weight, and location of tumors were recorded. Tumor tissue was snap-frozen for lysate preparation. The individuals who performed the necropsies, tumor collection, and tissue processing were blinded to treatment group assignments.

### Transgenic gastric cancer mice models

Gastritis and GC animal models were developed using K19-C2mE [Tg(Krt19-Ptgs2, Krt19-Ptges)8Tko] and Gan (K19-Wnt1/C2mE) [Tg(Krt19-Wnt1)2Maos/Tg(Krt19-Ptgs2, Krt19-Ptges)8Tko] transgenic mice, as described previously [14].

### Western blotting

Western blot analysis was performed as described previously [16]. After blocking the membrane with bovine serum albumin (BSA), anti-FAT1 (A304-403A; Bethyl Laboratories Inc.), anti-YAP1 (sc-500; Santa Cruz Biotechnology), anti-β-actin (A5316; Sigma-Aldrich), or anti-α-tubulin (#3873; CST) antibodies diluted 1:1000 in (BSA) were applied.

### shRNA silencing of FAT1

Small hairpin (sh) RNA vectors were purchased from Sigma infusion systems (targeting sequence: FAT1#156 (TRCN0000245156); CCGGAGTGATCTCAGTCCGTTTAATCTCGAGATTAAACGGACTGAGATCACTTTTTTG, FAT1#024 (TRCN0000038024); CCGGCCAGGGTTTCATTCTGTCTTTCTCGAGAAAGACAGAATGAAACCCTGGTTTTTG. To produce lentiviral particles, the vector was co-transfected using Lipofectamine 3000 (Invitrogen) with the lentiviral packaging and envelope plasmids psPAX2 and pMD2.G (Addgene) into 293FT cells. At 48–72 h post transfection. Pooled supernatants were concentrated using an Amicon Ultra-15 50K cutoff filer device (Millipore). Cells were transduced with the virus-containing medium at a moderate multiplicity of infection (MOI=3) and selected in 1 μg/ml puromycin. Experiments examining the effects of shRNA were performed 2–4 days after transduction.

### Organoid culture

The organoid culture protocol was as previously described [3] with minor modifications. Gastric fundus organoids were derived from surgical samples from GC patients from the Asan Medical Center Seoul, Korea (IRB No. S2017-1308-0001). GC tissue was mixed in Matrigel (BD Biosciences, San Jose, CA) in culture. The cell/Matrigel mixture was cultured in Advanced DMEM/F-12 medium (Invitrogen, Carlsbad, CA) containing Wnt-conditioned medium and R-spondin-conditioned medium supplemented with gastric growth factors including bone morphogenetic protein inhibitor, noggin (PeproTech, Rocky Hill, NJ), gastrin (Sigma, St Louis, MO), epidermal growth factor (PeproTech), and fibroblast growth factor 10. Cells matured into organoids after 1–2 days. Gastric organoids were passaged every 12 days.

### Organoid immunostaining

To identify the number of proliferating cells, GC organoids were incubated in 5 mM 5-ethynyl-2’-deoxyuridine (EdU; Invitrogen) in growth medium for 1 hour at 37°C. Organoids were fixed for 15 minutes with 4% para-formaldehyde in PBS at room temperature and then washed in 3% BSA in PBS twice for 5 minutes. Organoids were permeabilized using 0.5% Triton X-100 in PBS for 15 minutes at room temperature, followed by two washes in 3% BSA in PBS. The Click iT reaction cocktail (#C10340; Invitrogen) was added to the cells according to the manufacturer's instructions and incubated for 30 minutes at room temperature, followed by two washes in PBS. Organoids were then incubated with the DNA dye Hoechst 33342 (Invitrogen) at a dilution of 1:1000 in PBS for 30 minutes and washed twice with PBS. Fluorescence microscopy was performed using an LSM780 inverted confocal laser scanning microscope (LSM: Carl Zeiss, Jena, Germany). Total cell number and EdU-labeled nuclei were counted and expressed as a ratio of EdU-positive cells/ total cell number.

### Confocal laser scanning microscopy

Fluorescence microscopy was performed using an LSM780 (Carl Zeiss). LSM observations were all performed at room temperature. For dual color observation, EdU and FAT1 were detected by excitation at 647 nm using an argon laser (0.3% laser output) with a 650 to 670 nm long pass filter, and by excitation at 488 nm using a DPSS laser (0.3% laser output) with a 495 to 519 nm long pass filter, respectively. To avoid bleed-through effects in double-scanning experiments, EdU and FAT1 were scanned independently in multi-tracking mode. The z-stack profiles (total stack size, 80 μm) were acquired at 2 μm intervals from the bottom to the top of the organoid. Microscopy images were processed and analyzed using ZEN2012 software installed on the LSM780.

### Concave microwell migration and invasion assay

A recently developed concave microwell system [15], [20] was used to assay cell migration and invasion. A 0.1 ml suspension of AGS cells at a concentration of 2 × 10^6^ cells/ml was directly seeded on top of the concave microwell-arrayed polydimethyl siloxane (PDMS) plate with gentle shaking, and the cells were allowed to become trapped within the concave microwells. Thirty minutes after seeding, when most AGS cells had settled within the concave microwells, a flow of culture medium was gently applied to remove any remaining in suspension.

### Microfluidic chip device with collagen scaffold

To evaluate the effect of VP on cell migration, a microfluidic assay incorporating a hydrogel scaffold was fabricated as previously described [4], [9]. Briefly, the microfluidic devices were made of PDMS Sylgard 184 (Dow Chemical, MI, USA) patterned on an SU-8-patterned silicon wafer (MicroChem, MA, USA) using a conventional soft lithography process. The scaffold region was filled with type 1 collagen (BD BioSciences, MA, USA). After gelation, MKN45 cells (2 × 10^6^ cells/ml) were seeded into the microfluidic channel (center channel). After cell attachment, culture medium was introduced into all the channels (cell, control, and drug channels). One day after cell seeding, medium was refreshed and 5 μM VP was introduced into the drug channel.

### Statistical analysis and survival analysis

The random variance t-test was applied to identify genes differentially expressed between the two classes using BRB-ArrayTools (National Cancer Institute, Bethesda, MD). Gene expression differences were considered statistically significant if the p-value was <0.001. Cluster analysis was performed with Cluster and Treeview. Kaplan–Meier plots and log-rank tests were used to estimate patient prognosis.

## SUPPLEMENTARY MATERIALS FIGURES


