# Correction: Verteporfin inhibits gastric cancer cell growth by suppressing adhesion molecule FAT1

**DOI:** 10.18632/oncotarget.26488

**Published:** 2018-12-14

**Authors:** Myoung-Hee Kang, Gi Seok Jeong, Duane T. Smoot, Hassan Ashktorab, Chang Mo Hwang, Byung Sik Kim, Hee Sung Kim, Yun-Yong Park

**Affiliations:** ^1^ Asan Institute for Life Sciences, Asan Medical Center, Seoul, Republic of Korea; ^2^ Department of Convergence Medicine, University of Ulsan College of Medicine, Seoul, Republic of Korea; ^3^ Biomedical Engineering Research Center, Asan Institute for Life Sciences, Asan Medical Center, Seoul, Republic of Korea; ^4^ Department of Internal Medicine, Meharry Medical College, Nashville, TN, USA; ^5^ Department of Medicine and Cancer Center, Howard University, Washington, DC, USA; ^6^ Department of Gastric Surgery, University of Ulsan College of Medicine, Asan Medical Center, Seoul, Republic of Korea

**This article has been corrected:** In the Materials and Methods section, regarding the Gene expression profiling, the Correct number of Gene Expression Omnibus public database (GSE77982) as shown below:

**Gene expression profiling**

ASG or MKN45 cells were harvested for RNA isolation with or without treatment with VP for 48 hours. Total RNA was extracted using a mirVana miRNA isolation labeling kit (Ambion Inc., Waltham, MA), and 500 ng was used for labeling and hybridization (Human BeadChip V4 microarray, Illumina, San Diego, CA) according to the manufacturer’s protocols. After the bead chips were scanned with an Illumina BeadArray Reader, the microarray data were normalized using the quantile normalization method in the Linear Models for Microarray Data package in R (www.r-project.org). The expression level of each gene was log2 transformed before further analysis. The microarray data generated in this study are available in the NCBI Gene Expression Omnibus public database (GSE77982).

Original article: Oncotarget. 2017; 8:98887-98897. https://doi.org/10.18632/oncotarget.21946

