# Supplementary material for: Verteporfin inhibits gastric cancer cell growth by suppressing adhesion molecule FAT1
Source: Oncotarget. 2017 Oct 19;8(58):98887–97. doi: 10.18632/oncotarget.21946 (PMC5716775; doi:10.18632/oncotarget.21946)
Supplement: Supplementary file 1 [file oncotarget-08-98887-s001.pdf]

Verteporfin inhibits gastric cancer cell growth by suppressing adhesion molecule FAT1

SUPPLEMENTARY MATERIALS

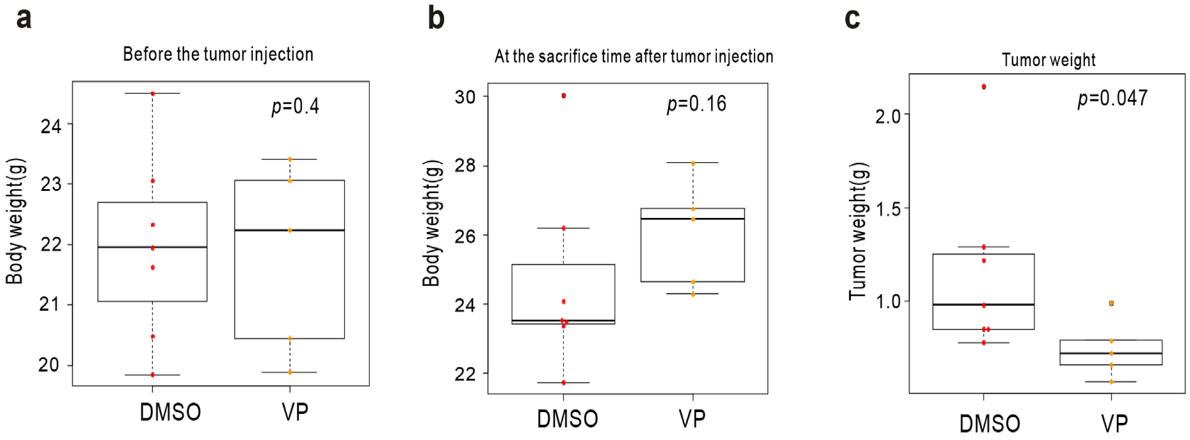

Supplementary Figure 1: VP treatment in xenograft mouse. (a-c) NCI-N87 GC cells were injected into nude mice with or without VP, and body weight and tumor volume was measured at the indicated time points. Mice were sacrificed and tumor volumes were measured. To see the significant difference between DMSO and VP, student *t-test* was used.

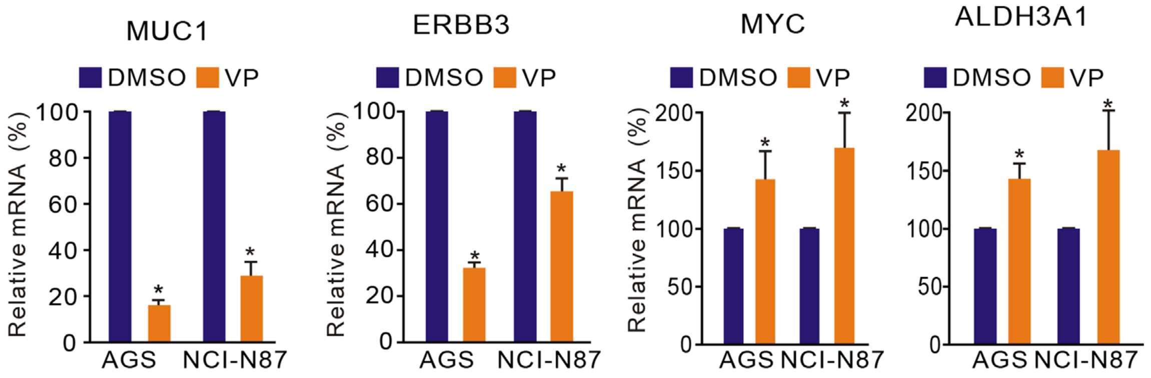

Supplementary Figure 2: Gene expression by VP treatment in gastric cancer cells. Indicated gastric can cells were treated with VP (3m M) for 48 hrs and the cell lysated were used for qRT-PCR analysis with indicated genes. Student t-test (two-tailed) was applied to estimate the significance of gene expression changes: *, $p < 0.001$.

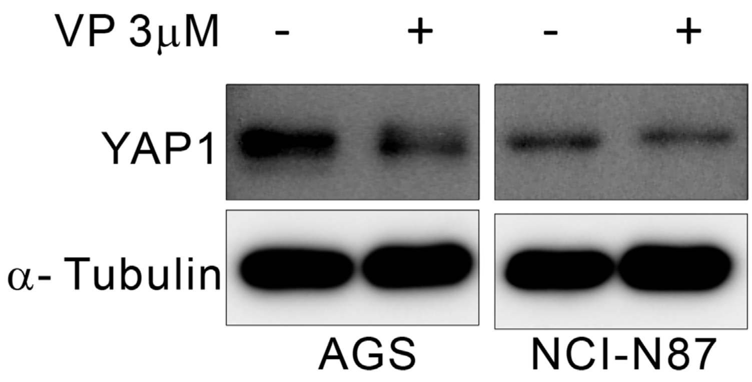

Supplementary Figure 3: YAP1 gene expression by VP in gastric cancer cells. YAP1 protein expression in indicated gastric cancer cells after VP treatment. The cell lysated were used for WB analysis with YAP1 antibody and a -tubulin was used as loading control.

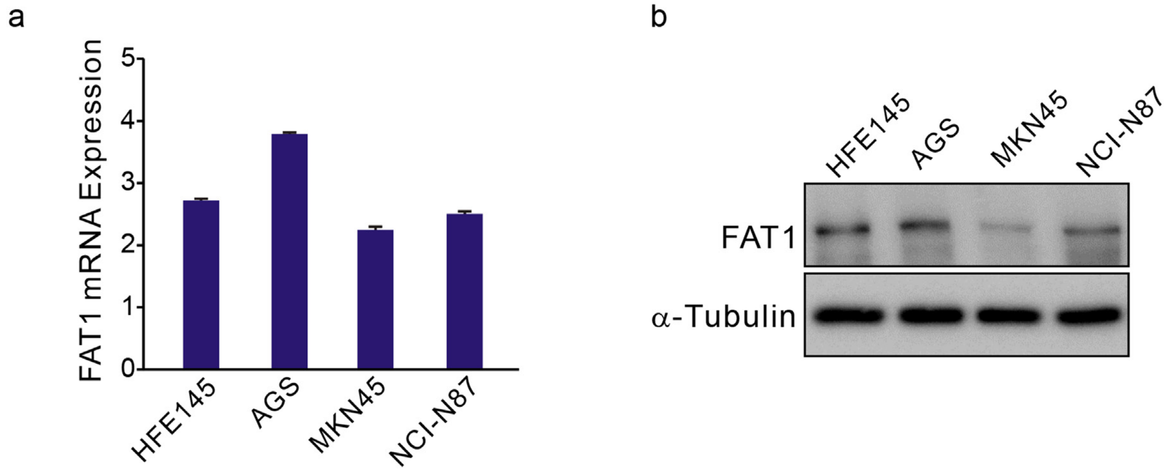

Supplementary Figure 4: FAT1 gene expression in gastric cancer cells in indicated gastric cancer cells. FAT1 mRNA **(a)** and protein expression **(b)** in indicated gastric cancer cells. The cell lysated were used for qRT-PCR with FAT1 probe and FAT1 expression level was normalized with PPIA genes. The cell lysated were also used used for western blots analysis to detect FAT1 protein level.

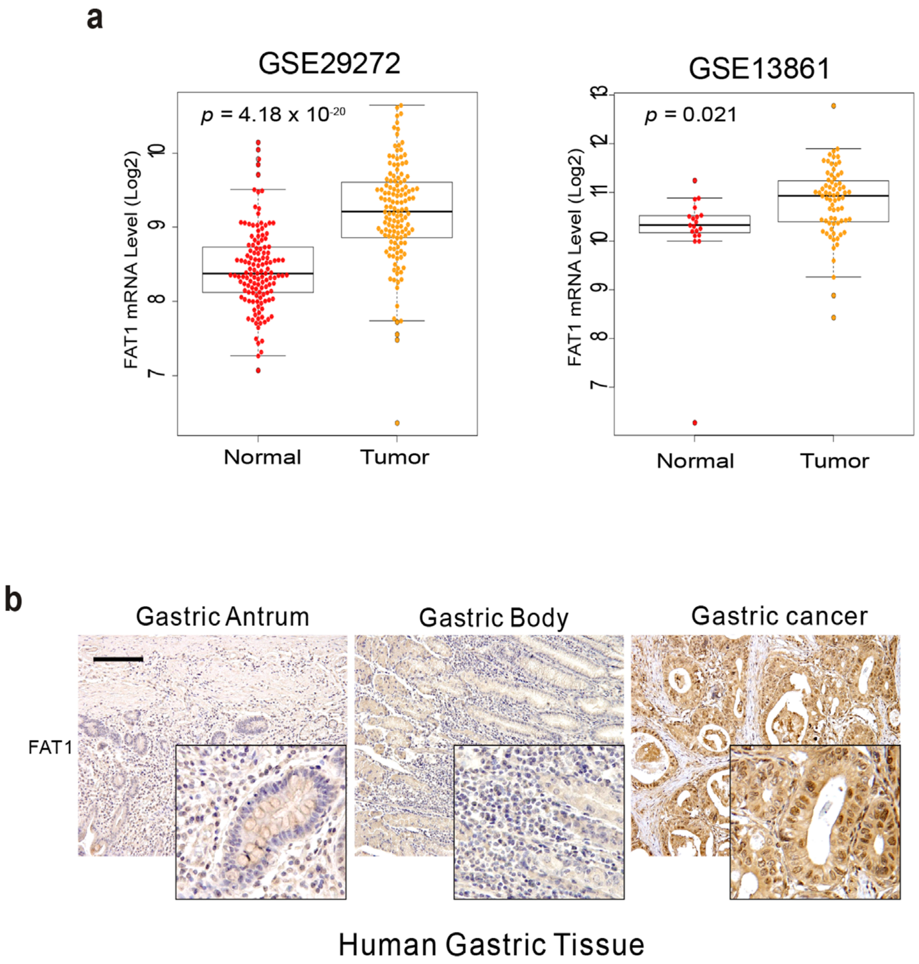

Supplementary Figure 5: FAT1 expression in human gastric cancer. **(a)** FAT1 expression in indicated gastric cancer cohort. The *p* values were calculated by Student *t*-test. **(b)** IHC form gastric cancer patients using FAT1 antibody.

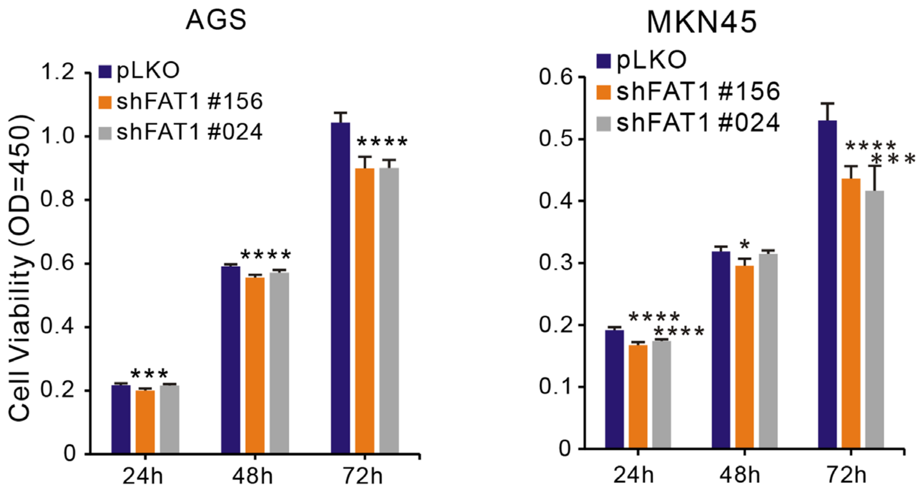

Supplementary Figure 6: Cell proliferation by FAT1 in gastric cancer cells. VP was treated to indicated gastric cancer cells and CCK8 was done at the indicated time. Student *t*-test (two-tailed) was applied to estimate the significance of gene expression changes: *, $p < 0.05$, **, $p < 0.01$***, $p < 0.005$, ****, $p < 0.001$.

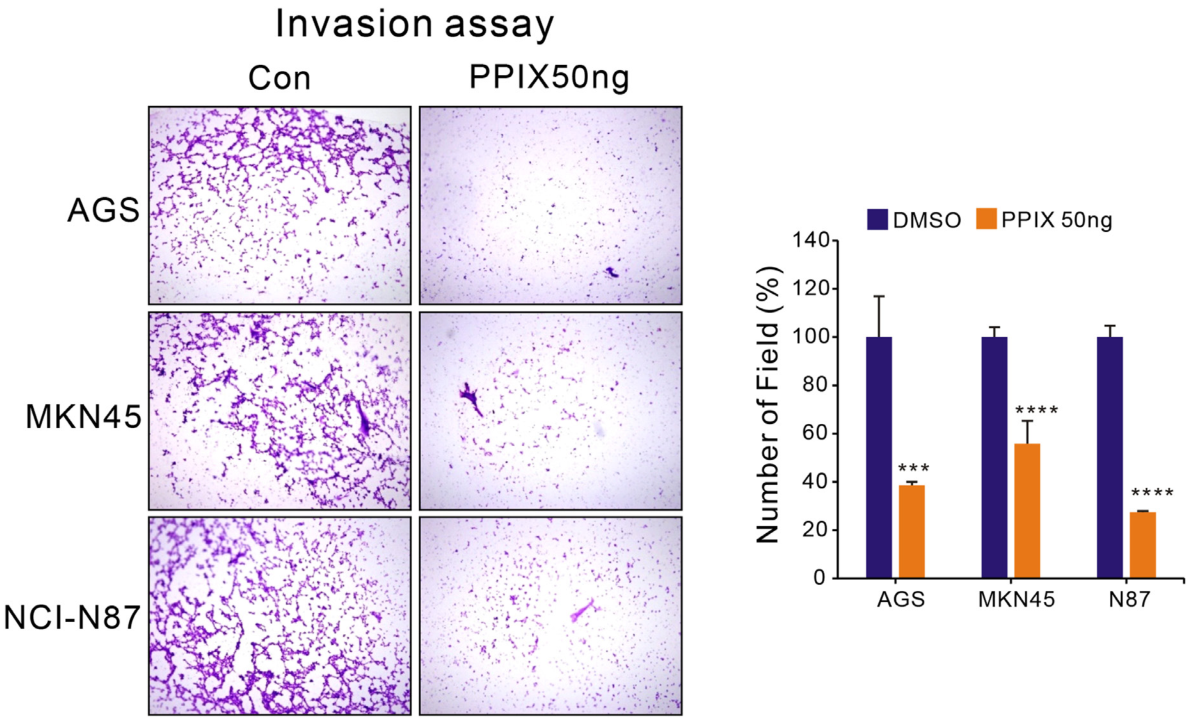

Supplementary Figure 7: PPIX effect in cell invasion Conventional cell invasion assay. Cells (1 x 10^5 cells/well) were seeded in the upper chamber, which was coated with Matrigel, and growth medium containing PPIX (50 ng) was added to the lower chamber. After 48 h of incubation, the cells that invaded the lower surface of the insert were stained with Diff-Quik stain and counted by microscopy. The *p* values were calculated by Student *t*-test. ***, $p<0.005$, ****, $p<0.001$.

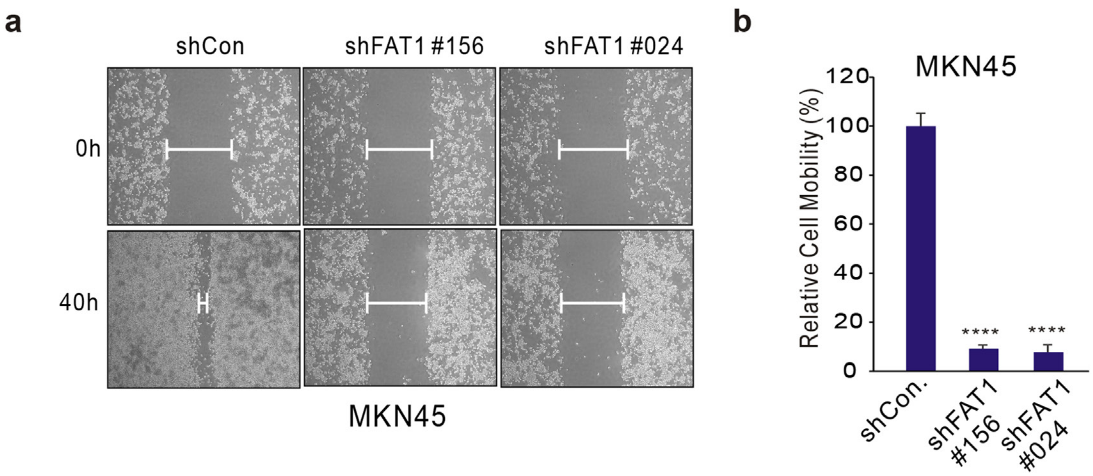

Supplementary Figure 8: FAT1 function in cell migration. **(a)** MKN45 cells were infected with shCon or shFAT1 and wound healing assay was done. **(b)** wound healing assay with infected cells was done. The *p* values were calculated by Student *t*-test. ****, $p<0.001$.

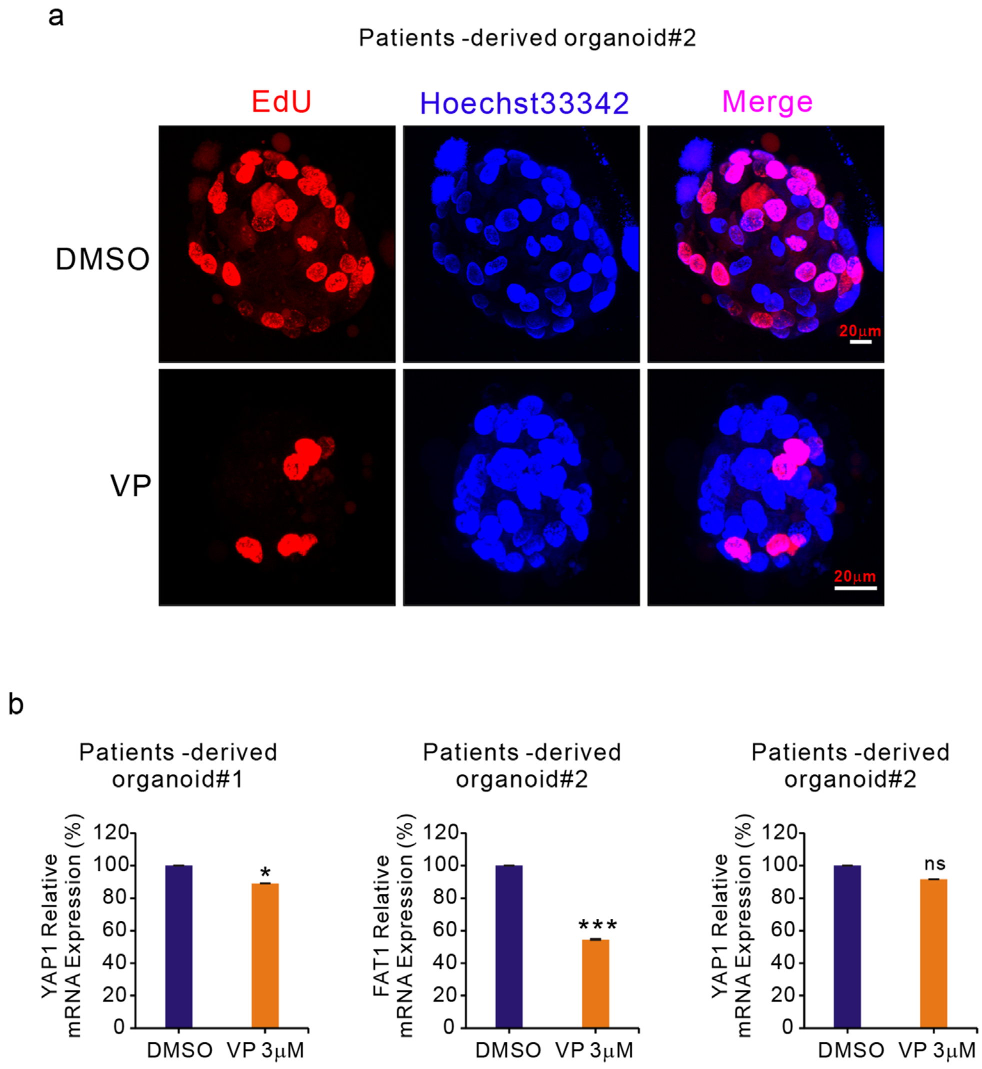

Supplementary Figure 9: Anti-proliferative effect of VP in human organoid. **(a)** Human organoid was isolated from gastric cancer patient. Isolated tissue was cultured following the protocol. Organoid was stained with EdU to measure the growth. **(b)** FAT1 and YAP1 expression level from the organoid using qRT-PCR. The *p* values were calculated by Student *t*-test. *, $p<0.05$, ***, $p<0.005$
